# The oligodeoxynucleotide sequences corresponding to never-expressed peptide motifs are mainly located in the non-coding strand

**DOI:** 10.1186/1471-2105-11-383

**Published:** 2010-07-20

**Authors:** Giovanni Capone, Giuseppe Novello, Candida Fasano, Brett Trost, Mik Bickis, Anthony Kusalik, Darja Kanduc

**Affiliations:** 1 Department of Biochemistry and Molecular Biology "Ernesto Quagliariello", University of Bari, Bari, Italy; 2 Department of Computer Science, University of Saskatchewan, Saskatoon, Canada; 3 Department of Mathematics and Statistics, University of Saskatchewan, Saskatoon, Canada

## Abstract

**Background:**

We study the usage of specific peptide platforms in protein composition. Using the pentapeptide as a unit of length, we find that in the universal proteome many pentapeptides are heavily repeated (even thousands of times), whereas some are quite rare, and a small number do not appear at all. To understand the physico-chemical-biological basis underlying peptide usage at the proteomic level, in this study we analyse the energetic costs for the synthesis of rare and never-expressed versus frequent pentapeptides. In addition, we explore residue bulkiness, hydrophobicity, and codon number as factors able to modulate specific peptide frequencies. Then, the possible influence of amino acid composition is investigated in zero- and high-frequency pentapeptide sets by analysing the frequencies of the corresponding inverse-sequence pentapeptides. As a final step, we analyse the pentadecamer oligodeoxynucleotide sequences corresponding to the never-expressed pentapeptides.

**Results:**

We find that only DNA context-dependent constraints (such as oligodeoxynucleotide sequence location in the minus strand, introns, pseudogenes, frameshifts, etc.) provide a coherent mechanistic platform to explain the occurrence of never-expressed versus frequent pentapeptides in the protein world.

**Conclusions:**

This study is of importance in cell biology. Indeed, the rarity (or lack of expression) of specific 5-mer peptide modules implies the rarity (or lack of expression) of the corresponding *n*-mer peptide sequences (with *n *< 5), so possibly modulating protein compositional trends. Moreover the data might further our understanding of the role exerted by rare pentapeptide modules as critical biological effectors in protein-protein interactions.

## Background

Proteins comprise subsets of all plausible amino acid sequences, i.e. peptide motifs that occur in different quantitative percentages and with different qualitative significance at the proteomic level. To understand the correspondence between structure and function, we must understand the rules dictating the modular arrangement of proteins. We chose the pentapeptide as a basic structural/functional unit to analyse the compositional distribution of peptide sequences. Indeed, pentapeptides appear to be minimal biological units exerting a central role in fundamental cellular processes such as inhibition/stimulation of cell growth, hormone activity, regulation of transcript expression, enzyme activity, and immune recognition [1]. Following a robust set of experimental protein analyses [2-9], we determined that, as a rule, amino acid stretches with low/no proteomic redundancy alternate with portions of high proteomic redundancy along protein primary structures [2], independently of the protein length [3,4], whether the protein is derived from microbial or mammalian organisms [3-9], and the proteome under analysis [5-9]. Preliminarily to any evolutionary/functional/physio-pathological considerations, the data prompt a fundamental question: what makes one pentapeptide occur more frequently than another in the protein world? In this paper, we undertake a large-scale analysis of the physico-(bio)chemical factors that theoretically might account for the modular peptide composition of proteins, and examine a total of 20991 pentapeptides, divided into eleven sets characterized by frequencies ranging from zero to 2500.

## Methods

The complete UniRef100, UniRef90 and UniRef50 databases (http://www.uniprot.org/downloads) were downloaded as single proteomes and analysed for internal peptide redundancy using 5-mers sequentially overlapping by four residues. The scans were performed using standard UNIX/LINUX commands and custom programs written in Perl [10].

The proteins were manipulated and analysed as follows. All the protein sequences were decomposed *in silico *to a set of 5-mers (including all duplicates). Any 5-mers containing ambiguous amino acids (i.e., denoted by the letters B, X, or Z, which respectively represent ambiguity between N and D, ambiguity between Q and E, and an unknown amino acid) or non-standard amino acid codes (i.e., -, U, *, O, denoting gaps, selenocysteine residues, stop codons, etc.) were eliminated. Since there are only 3200000 possible 5-mers, a simple linear scan was used to determine the counts of occurrences and 5-mers that do not occur. That is, for each pentamer, the UniRef100 (or UniRef90 or UniRef50) proteome was searched for instances of that pentamer. Any such occurrence was termed a match. The number of matches defines the proteomic frequency of each pentapeptide.

Eleven peptide sets with zero, low, medium and high frequencies (i.e., from zero to 2500 matches) were selected from UniRef100 (hereafter called the "universal proteome") for physico-(bio)chemical analyses. Specifically, the frequencies defining the eleven sets were: 0, 1, 4, 5, 50, 100, 341, 500, 1000, 1368 and 2500. The pentapeptide sets were screened by starting with the UniRef100 database and then using the Perfect Peptide Match program at the Protein Information Resource (PIR) website (http://pir.georgetown.edu/pirwww) [11] to eliminate repeated sequences and fragments. The protein entries containing the 5-mer under analysis were further filtered using the UniProtKB resources (http://www.uniprot.org) to eliminate obsolete entries.

Analysis of the energetics was carried out for each pentaptide using Spartan'06 software (from Wavefunction Inc, Irvine, CA) and applying the semi-empirical method. The peptide bulkiness degree was measured using the ProtScale program available at http://www.expasy.ch/tools[12]. The hydrophobicity level was determined using the scale described by Takano and Yutani [13]. The codon number per pentapeptide was calculated by summing the number of codons of each amino acid forming the 5-mer. One-way analysis of variance (ANOVA, F-test) was used to derive a p-value indicating whether the means of the measurements for the different sets were all equal.

To analyse DNA constraints, we analysed the oligodeoxynucleotide coding sequences corresponding to the pentameric amino acid sequences. The Sequence Manipulation Suite Reverse Translate program (http://www.bioinformatics.org/sms2/) [14] was used to generate a DNA sequence representing the most likely, optimized coding sequence. Additionally, Reverse Translate a Protein (http://www.vivo.colostate.edu/molkit/rtranslate/index.html), a program that uses the standard genetic code and does not consider differences in codon usage, was used in order to obtain all the possible degenerate oligodeoxynucleotide coding frames for each pentapeptide under analysis.

The pentadecameric oligodeoxynucleotide sequences so obtained were the subject of nucleotide-nucleotide BLAST (blastn) analysis at NCBI (http://blast.ncbi.nlm.nih.gov) to find and localize regions of 100% similarity (i.e. with no gaps allowed) in the entire nucleotide collection (nr/nt) comprehending genomic and transcript sequences [15].

## Results

### Pentapeptide redundancy and ΔG°

The biosynthesis of the peptide bond from amino acids involves an increase in free energy and must therefore depend on energy yielding reactions. We reasoned that, if a substantial fraction of energy is needed to convert starting amino acids into peptides, then the pentapeptide composition of proteins expressed in the proteomes should be biased toward less energetically costly pentapeptides. Theoretically, the extent to which pentapeptide composition is biased to reduce metabolic costs should positively correlate with the pentapeptide redundancy at the proteomic level.

**Figure 1:**
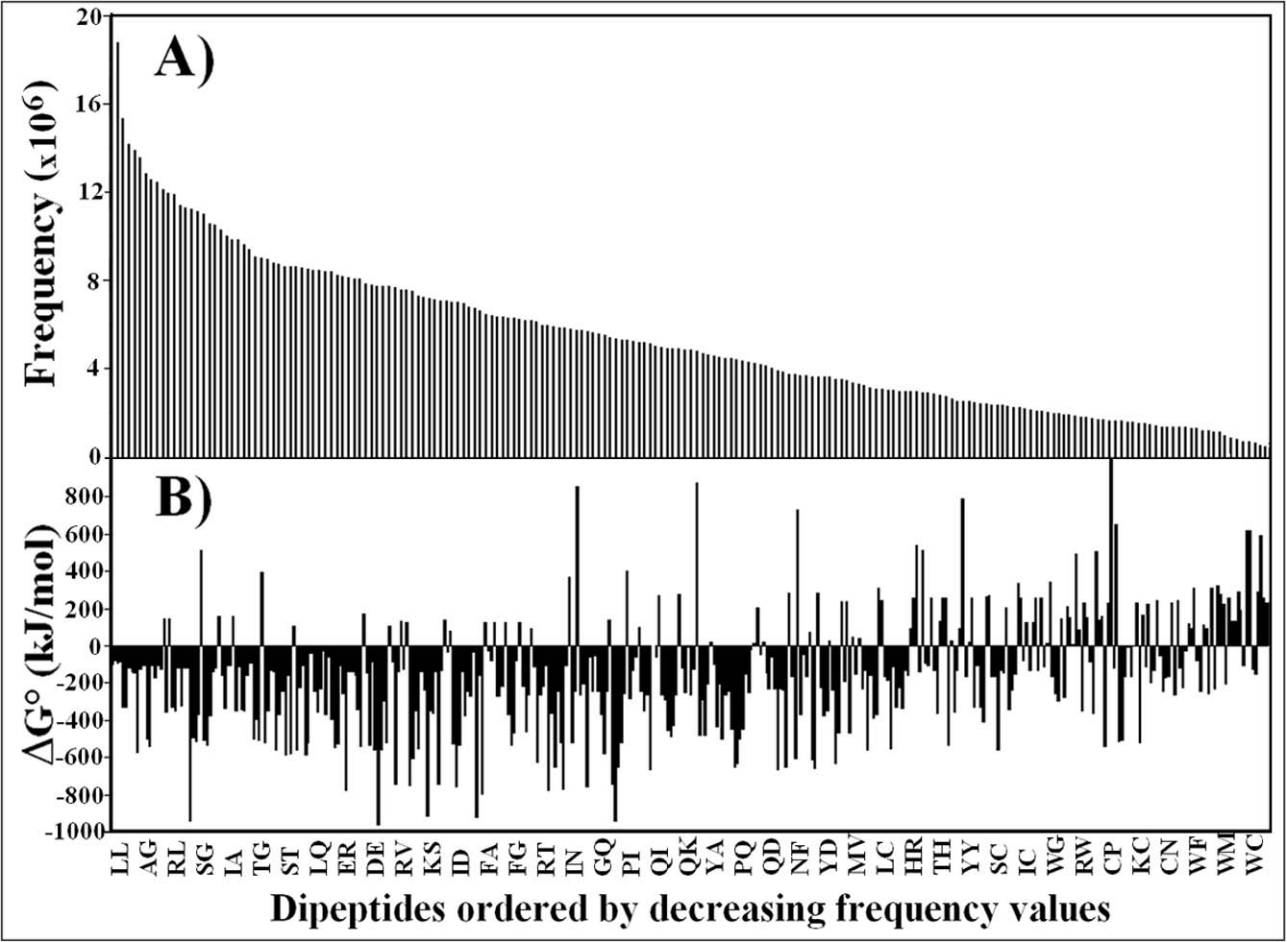
**Correlation between frequency distribution and standard heat of peptide formation of the 400 dipeptides present in the protein world**. Panel A: The frequency distribution of the 400 dipeptides present in the protein world widely varies from a maximum of 21,927,296 times (LL dipeptide) to a minimum of 407,573 (WC dipeptide). Panel B: The heat of formation in kJ/mol as determined by the Spartan'06 software for the 400 dipeptides varies widely from the highly exothermic value of DE dipeptide formation (-944.34 kJ/mol) to the endothermic CP dipeptide formation (1062.47 kJ/mol). Mean ΔG° values equal to -219.06 ± 237.83 kJ/mol and 56.34 ± 249.51 kJ/mol characterize the 50 most frequent dipeptides and the 50 less frequent ones, respectively.

Consequently, we analysed rare versus frequent pentapeptides for the standard enthalpy (or standard heat of formation) associated with the synthesis of the peptide bond. This quantity is highly variable, with the heat generated or absorbed during the formation of a peptide bond depending on the amino acids involved. As an example, Figure 1A reports the frequency distribution of the 400 dipeptides present in the protein world, and, in parallel, the heat of formation in kJ/mol as determined using Spartan '06 software (Figure 1B). It can be seen that the standard heat of formation of the semi-empirically optimised dipeptide structures varies widely from the highly exothermic value of DE dipeptide formation (-944.34 kJ/mol) to the endothermic CP dipeptide formation (1062.47 kJ/mol) (Figure 1B). Moreover, Figure 1 shows that a negative correlation exists between dipeptide redundancy (Figure 1A) and ΔG° level (Figure 1B). As a synthetic datum, mean ΔG° values equal to -219.06 ± 237.83 kJ/mol and 56.34 ± 249.51 kJ/mol characterize the 50 most frequent dipeptides and the 50 less frequent ones, respectively.

Therefore, we reasoned that the same ΔG° variability would apply even more strongly to longer peptide units. Based on this rationale, we calculated the heats of formation for pentapeptide sets with different frequencies in the universal proteome (i.e., from zero to 2500 occurrences). As a universal proteome database, we used UniRef100, which represents one of the most comprehensive non-redundant protein sequence datasets available ([16-18], see also http://www.ebi.ac.uk/uniref/). To control for existing bias and redundancies in the UniRef100 database, the protein entries containing the 5-mers under analysis were filtered for repeated sequences, fragments, and obsolete entries.

**Figure 2:**
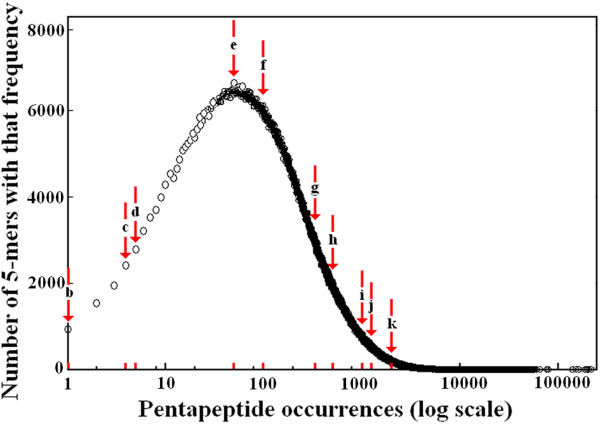
**Location of the 5-mer sets selected for physico-chemical analyses along the distribution curve of pentapeptide frequencies in the universal proteome**. UniRef100, the most comprehensive protein dataset available [[16-18], see also http://www.ebi.ac.uk/uniref/], was used. The arrows, lettered from b to k, indicate the frequencies of the different 5-mer sets corresponding, in the order, to 1, 4, 5, 50, 100, 341, 500, 1000, 1368 and 2500 occurrences and selected for physico-chemical analyses. A further set a, corresponding to the set of never-occurring pentapeptides, was also chosen.

Figure 2 reports the distribution of pentapeptide frequencies in the universal proteome as the log of the occurrence count versus the number of 5-mers with that count. The same trend in the quantitative pentapeptide composition of the protein world was observed using UniRef90 and UniRef50 protein datasets (not shown). Then, we selected pentapeptide sets for physico-chemical analyses along the distribution curve of pentapeptide frequencies shown in Figure 2. The frequencies of the different 5-mer sets elected for analysis correspond to 1, 4, 5, 50, 100, 341, 500, 1000, 1368 and 2500 occurrences (as indicated by the lettered arrows b to k, plus a 5-mer set having zero occurrences, namely a). That is, we selected: peptides occurring just once for the obvious reason that such peptides are expected to be "interesting"; the occurrence count of 50 was chosen because the maximum is reached at this point; 341 was chosen because it is a median value (i.e. half the peptides have occurrence counts less than this value, and half the peptides have occurrence counts more than this value); high occurrence counts (e.g. 2500) were chosen to represent the "tail" of the distribution. However, extremely high counts (e.g. 5000 or more) were not chosen because the number of pentapeptides with these frequencies tended to be too small to give results in which we were confident. Finally, other occurrence counts were chosen so as to broaden this sampling.

**Figure 3:**
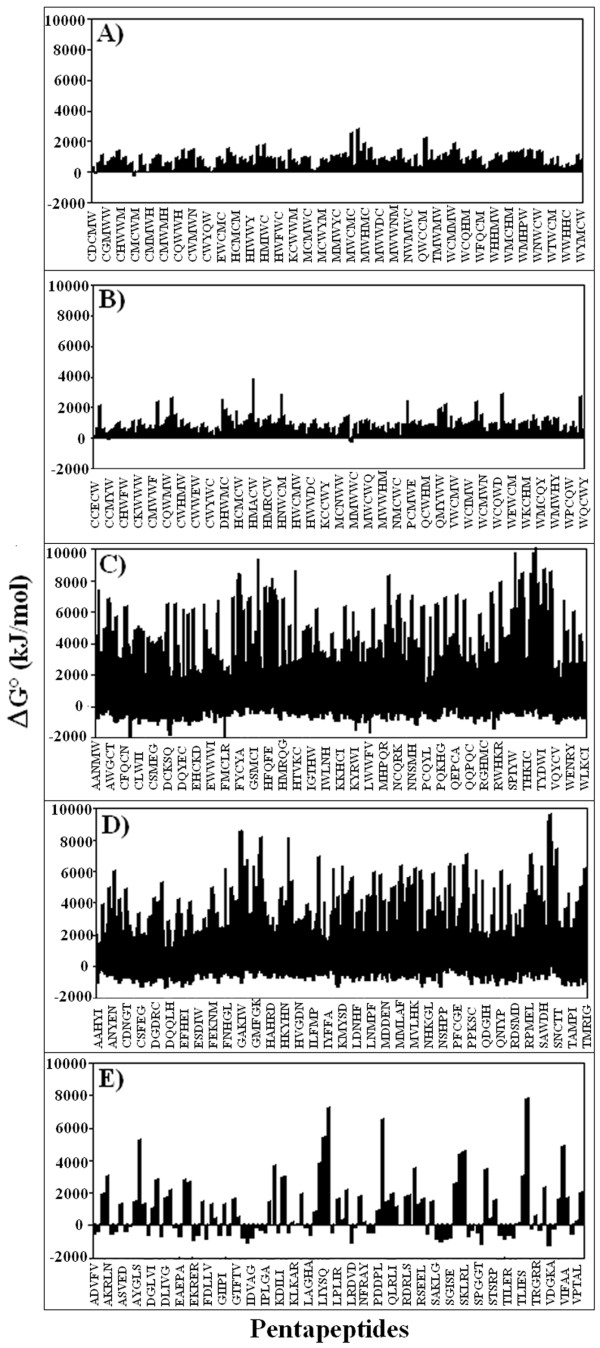
**Energetic cost of pentapeptides with different frequencies in the universal proteome**. Panels A to E indicate pentapeptide sets that, in the universal proteome: A) are absent, B) are expressed only once, C) occur 100 times, D) occur 341 times, and E) occur 2500 times.

Afterwards, we calculated the relationship between metabolic costs of pentapeptide biosynthesis (as estimated from heat of peptide bond formation data) and pentapeptide redundancy (as estimated by the number of occurrences). The histograms reported in Figure 3, Panels A to E, refer to the energetic profiles of pentapeptide sets with the following different frequencies in the universal proteome: A) never expressed, B) expressed only once, C) occurring 100 times, D) occurring 341 times, and E) occurring 2500 times. It can be seen that the range in heat of formation values varies considerably across the five sets of pentapeptides. For instance, many among the high frequency pentapeptides have extremely high or low heat-of-formation values (panels C, D and E), while the absent or rare pentapeptides fall into an energetically narrower window (panels A and B). Because of this large variance, considering the central tendency in each panel does not seem to allow one to distinguish among the sets.

**Figure 4:**
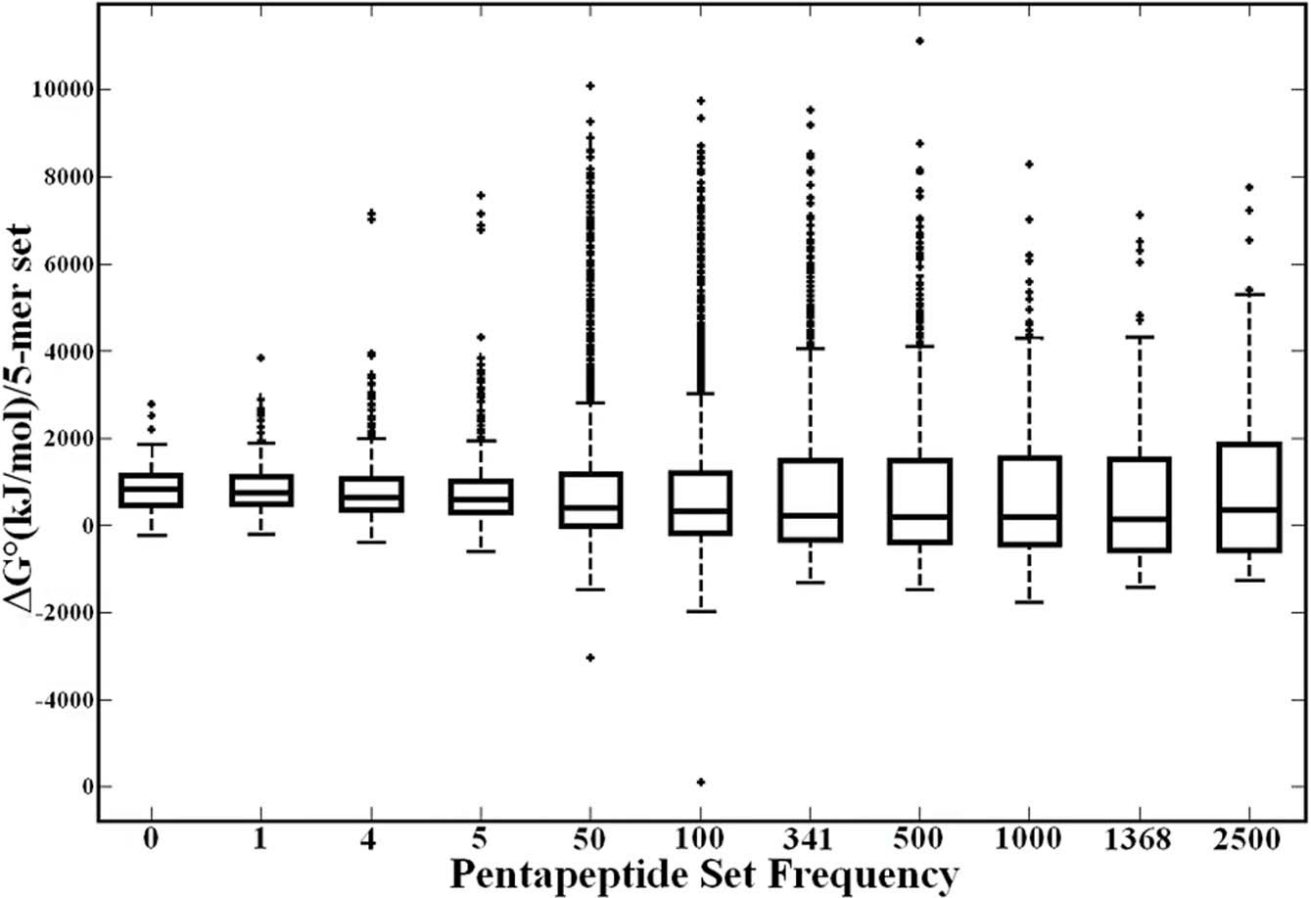
**Statistical characterization of the energetic cost of pentapeptide sets with different frequencies in the universal proteome**. The boxplots show the distribution of ΔG° values for each set of pentapeptides. The line within each box represents the median value. The top and bottom of each box represent the 75th and 25th percentile, respectively. The whiskers show the range of values that are not considered to be outliers. Outliers are plotted individually as plus signs. The p-value was 0.008, indicating that the means of the different sets are different, though clearly the magnitude of the differences is small.

This result is even more clear in the boxplot diagram reported in Figure 4, where the analysis of the distribution of ΔG° scores is extended to eleven pentapeptide sets occurring with different frequencies in the universal proteome (see Figure 2). It is evident that the never-expressed pentapeptides are confined to restricted energy levels, i.e. have smaller variance, while, on the contrary, many of the pentapeptides occurring repeatedly in the universal proteome have higher energetic costs. Moreover, specifically and importantly, the boxplot diagram shows that outliers are usually associated with high frequency pentapeptides rather than rare ones. Figure 4 clearly shows that the heat of formation has no stringent influence on pentapeptide frequency.

### The relationship between pentapeptide redundancy and hydrophobicity, bulkiness, and codon number

**Figure 5:**
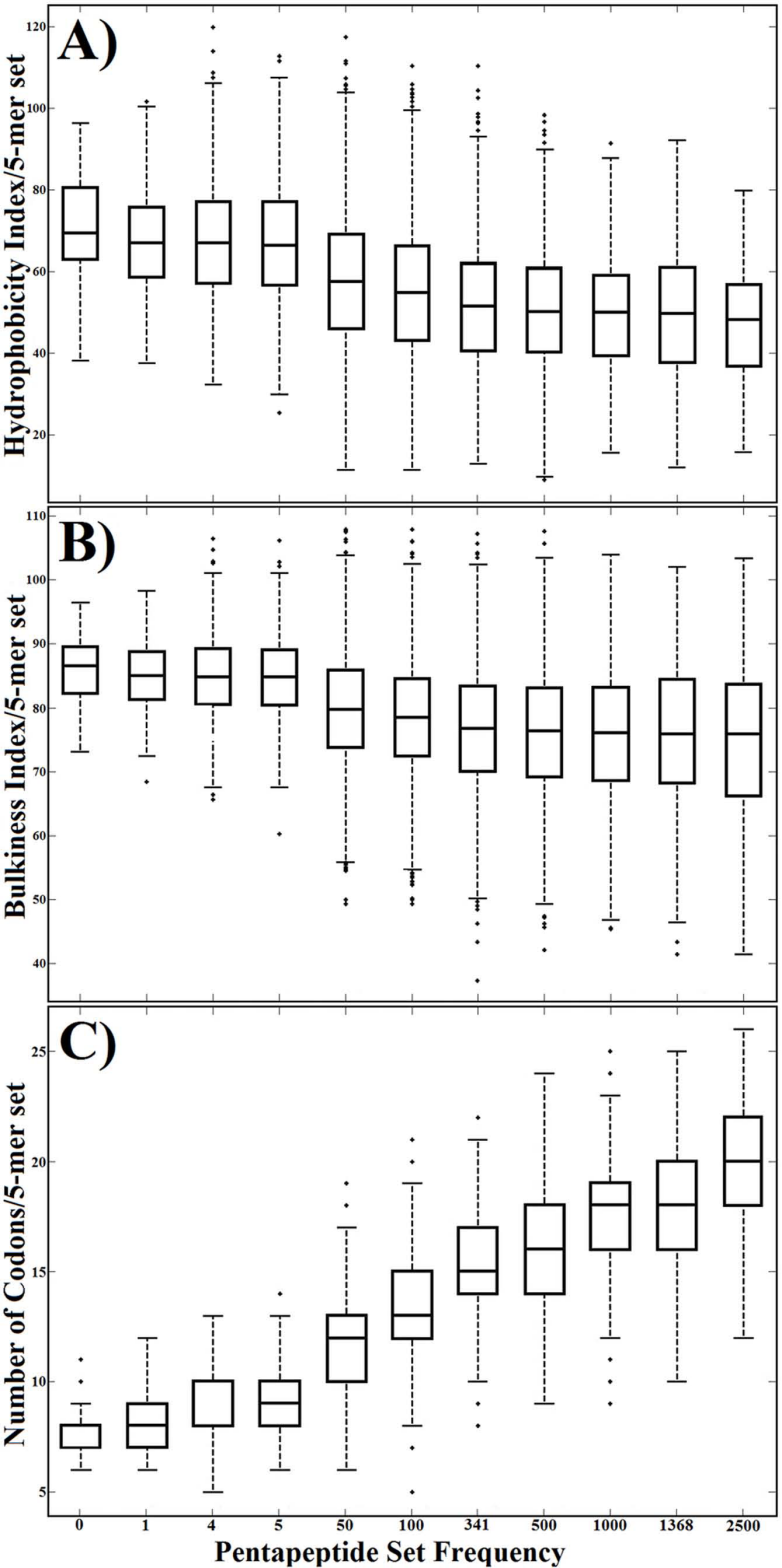
**Statistical characterization of hydrophobicity (A), bulkiness (B), and amino acid codon number (C) for pentapeptide sets with different frequencies in the universal proteome**. The boxplots show the distribution of the values of each physico-biochemical factor for each set of pentapeptides. The line within each box represents the median value. The top and bottom of each box represent the 75th and 25th percentile, respectively. The whiskers show the range of values that are not considered to be outliers. Outliers are plotted individually as plus signs. The p-values among the different classes of 5-mers for hydrophobicity, bulkiness, and amino acid codon number were all less than 0.001, indicating in each case that the means of the different sets are different.

We analysed the relationship between pentapeptide frequencies and the following physico-(bio)chemical parameters: side-chain bulkiness, hydrophobicity and amino acid codon number. The results are reported in Figure 5: it can be seen that the pentapeptide redundancy appears to be shaped by, in order of importance, the amino acid codon number (panel C), residue hydrophobicity (panel A), and residue bulkiness (panel B). However, in all instances many values are outliers, indicating a non-stringent relationship between the physico-chemical factors analysed and the distribution of pentapeptide redundancy in the universal proteome.

### Pentapeptide redundancy and amino acid composition

Figures 4 and 5 indicate almost no relationship between pentapeptide frequencies and physico-chemical factors such as hydrophobicity and bulkiness. On the other hand, the analyses reported in Figure 3 suggest that rare pentapeptides are formed primarily by Trp, Tyr, and Met, i.e. by essential low-concentration amino acids endowed with high values of hydrophobicity and residue bulkiness. This raises the question: might amino acid frequencies affect pentapeptide frequency?

**Figure 6:**
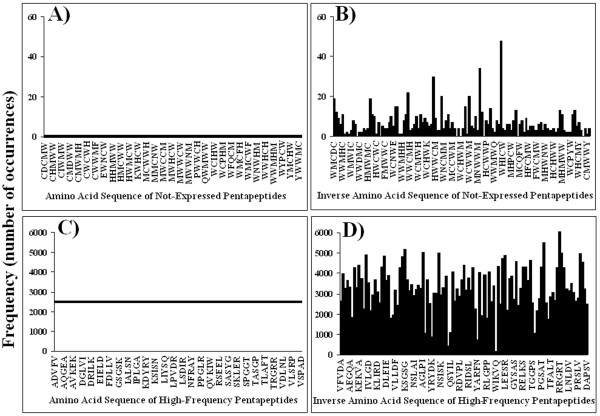
**Effect of amino acid composition on pentapeptide frequency in the universal proteome**. Frequency of: A) pentapeptides never-expressed in the universal proteome, and B) their inverse sequences. Frequency of: C) pentapeptides with 2500 occurrences in the proteome, and D) their inverse sequences.

To analyse the relationship between pentapeptide frequency and amino acid composition, we used the pentapeptide set with zero occurrences and investigated the frequency of the corresponding inverse amino acid sequences. We reasoned that if the factor dictating the rarity/frequency of a certain pentapeptide was specific amino acid composition, then inverting the order of those amino acids but keeping constant the amino acid composition would have little or no effect on pentapeptide occurrence. Panels A and B of Figure 6 show that the inverse sequences of the never-expressed pentapeptides occur in the universal proteome as many as 50 times. Hence, amino acid composition does not represent the factor precluding the expression of the zero-frequency pentapeptide set. Similar results were obtained using the set of pentapeptides with 2500 occurrences in the universal proteome: Figure 6 panel D shows that the inverse amino acid sequences occur in the universal proteome with a wide variety of frequencies. As a further control the frequency of pentapeptides uniquely formed by the rare W, Y and M amino acids was determined. We found that the highly structured WWWWW, YYYYY and MMMMM pentapeptides occur 112, 972 and 1568 times, respectively, in the universal proteome. I.e., pentapeptides formed by rare, mono-codonic, highly hydrophobic, and bulky amino acid residues can even fall in the category of the "highly repeated" pentamers.

Taken together, these data indicate that amino acid composition appears to modulate at some extent, but does not dictate, the pentapeptide composition of the universal proteome.

### Analysing the never-expressed pentapeptides at the DNA level

After obtaining the results above, we postulated that the lack of occurrence of the pentapeptides never found in the universal proteome could be ascribed to a lack of the corresponding pentadecameric oligodeoxynucleotides in the DNA coding sequence. Therefore, a search was conducted for occurrences of the oligodeoxynucleotide sequences coding for the pentapeptides never expressed in the universal proteome using the standard nucleotide-nucleotide BLAST (blastn) program as described under Methods.

The two examples reported in Table 1 synthetically illustrate that while all of the pentadecameric oligodeoxynucleotide sequences corresponding to the zero-frequency pentapeptides are present in a number of different organisms, they are mainly located in the DNA minus strand, introns, frameshifts, or pseudogenes, i.e. in untranslatable DNA positions/structures. The data from Table 1 are further confirmed by the data given in Additional file 1, Table S1, where analysis of the most likely and degenerate oligodeoxynucleotide coding frames for each pentapeptide sequence is reported.

**Table 1:** The oligodeoxynucleotide sequences corresponding to never-expressed peptide motifs are mainly located in the non-coding strand

Organisms hosting the ATGTGGCATATGTGC oligodeoxynucleotide coding for MWHMC pentapeptide:						
**Taxonomic ID**	**Organism**	**Location of the oligodeoxynucleotide:**				

		**DNA minus strand**	**Intron**	**Pseudogene**	**Frameshift**	**UTRs**

293826	*Alkaliphilus metalliredigens *(1)	+				
491915	*Anoxybacillus flavithermus *(1)	+				
290318	*Chlorobium phaeovibrioides *(1)				+	
7719	*Ciona intestinalis *(1)					+
37769	*Cryptococcus bacillisporus *(1)	+				
7955	*Danio rerio *(1)				+	
352472	*Dictyostelium discoideum *(1)	+				
7220	*Drosophila erecta *(1)	+				
7227	*Drosophila melanogaster *(1)	+				
7238	*Drosophila sechellia *(1)	+				
7240	*Drosophila simulans *(1)	+				
7245	*Drosophila yakuba *(2)	+			+	
9595	*Gorilla gorilla gorilla *(1)	+				
9606	*Homo sapiens *(4)	+++		+		
9544	*Macaca mulatta *(1)	+				
10090	*Mus musculus *(2)	++				
39947	*Oryza sativa Japonica *(3)	++	+			
1308	*Streptococcus thermophilus *(2)	+			+	
9823	*Sus scrofa *(1)	+				
377629	*Teredinibacter turnerae *(1)	+				
296543	*Thalassiosira pseudonana *(1)	+				

**Organisms hosting the TGGTTTCAGTGCATG oligodeoxynucleotide coding for WFQCM pentapeptide:**						

**Taxonomic ID**	**Organism**	**Location of the oligodeoxynucleotide:**				

		**DNA minus strand**	**Intron**	**Pseudogene**	**Frameshift**	**UTRs**

315750	*Bacillus pumilus *(1)					+
3708	*Brassica napus *(1)	+				
485918	*Chitinophaga pineni *(1)	+				
8330	*Cynops pyrrhogaster *(1)	+				
7955	*Danio rerio *(3)	++				+
9685	*Felis catus *(1)	+				
69293	*Gasterosteus aculeatus *(1)	+				
233412	*Haemophilus ducreyi *(1)				+	
9606	*Homo sapiens *(7)	+++++++				
284590	*Kluyveromyces lactis *(2)	+			+	
9544	*Macaca mulatta *(1)	+				
269797	*Methanosarcina barkeri *(1)	+				
10090	*Mus musculus *(5)	+++++				
7955	*Nicotiana plumbaginifolia *(1)	+				
9598	*Pan troglodytes *(1)	+				
500485	*Penicillium chrysogenum *(2)	+				+
3988	*Ricinus communis *(1)				+	
29760	*Vitis vinifera *(2)	+				+
8364	*Xenopus tropicalis *(1)	+				

The never-expressed MWHMC and WFQCM pentapeptides are reported as examples. Pentapeptide sequences were retrotranslated into the most likely pentadecameric oligodeoxynucleotide coding sequences. Then, each pentadecameric oligodeoxynucleotide sequence was used as a probe to scan the entire NCBI nucleotide collection for exact pentadecameric matches using BLAST (blastn) program with no gaps allowed. The organism hosting the oligodeoxynucleotide sequence(s) is identified by its taxonomic identification number and latin name. Plus sign(s) indicate the oligodeoxynucleotide location(s) in the DNA. Abbreviation: UTRs, untranslated regions. Total number of oligodeoxynucleotide sequence occurrences is in parentheses. See also Additional file 1, Table S1.

From this we conclude that DNA context-dependent constraints (e.g., oligodeoxynucleotide sequence location in the minus strand, introns, splicing-dependent frameshifts, etc.) are the main factors limiting/preventing the expression of the corresponding amino acid sequences in the universal proteome.

## Discussion

The factors acting on the amino acid composition of proteins have been thoroughly investigated with particular attention to the habitat of the organisms (e.g., growth temperature and salinity) [19-22], sub-cellular localization (e.g., cytosolic, membrane or nuclear) [23], physical properties (e.g., mass and charge) [24], translational constraints [25], and the metabolic costs of amino acid biosynthesis [26]. In contrast, less attention has been dedicated to the structural and functional constraints acting on the peptide composition of proteins. Clearly, the empirical distribution of pentapeptide frequencies has, one way or another, an impact upon protein expression as well as on function/structure, and it is important to understand and define the physico-chemical-biological factors that correlate with pentapeptide frequencies in the protein world.

We already reported preliminary data showing that certain short sequences of amino acids (i.e. pentapeptides) are very common, whereas some are quite rare, and a small number do not appear at all in the collection of all known proteins [27]. Here we report the results of a comprehensive study of the influence of physico-(bio)chemical parameters (energetic cost, bulkiness, hydrophobicity and amino acid codon number), amino acid composition, and DNA constraints on pentapeptide expression in the protein world.

First, we observe a definite (although not determining) role of, in descending order of importance, amino acid codon number, hydrophobicity and bulkiness in modulating pentapeptide frequency in the universal proteome. On the other hand, we find that ΔG° has little influence in defining the pentapeptide composition of the universal proteome. This result is relevant and deserves to be emphasized. We explored in detail whether variations in the peptide bond energetical cost might explain the extent of the pentapeptide compositional bias in the universal proteome based on the following rationale. The data reported for protein amino acid composition indicate increases in the abundance of less energetically costly amino acids in highly expressed proteins [26]. Accordingly and further supported by the correlation existing between dipeptide redundancy (Figure 1A) and ΔG° level (Figure 1B), we expected that energetically costly pentapeptides would be rare, whereas more frequent pentapeptides would have a low energetic cost. In conflict with this theoretical expectation, the experimental data obtained in this study and reported in Figures 3 and 4 clearly demonstrate that there is no such correlation at the pentapeptide level. Surprisingly we found that high energies of formation are associated with moderately or highly frequent pentapeptides.

A second unexpected finding is that amino acid composition is a marginal factor in determining pentapeptide rarity: although enriched in hydropathic, rare amino acids such as Trp, Tyr, and Met, the inverse sequences of never-expressed pentapeptides are indeed expressed in the universal proteome.

Third, and as a logical consequence of the previous two points, we show that the constraints acting on pentapeptide expression mainly lie at the nucleotide sequence level. Once we excluded possible limitations due to Trp, Met, and Tyr rarity [28] (see Figure 6), we had to suppose that other constraints are active in defining the proteomic pentapeptide frequencies. Effectively, as demonstrated in Table 1 (see also Additional file 1, Table S1), we found that never-expressed pentapeptides correspond to untranslatable, frameshifted or mistranslated oligodeoxynucleotide sequences. In other words, allocation of the coding oligodeoxynucleotide in pseudogenes/minus strand/untranslated regions/introns as well the shift of the reading frame are the main factors determining the distribution of pentapeptide frequencies throughout the protein world.

## Conclusions

The results above are of importance both in the biochemical and functional cellular context. Indeed, as already described [1,3-10,29], it seems that rare pentapeptides are basic to control functions [30], whereas possibly frequent modules are preferentially involved in structure definition. In this regard, it is worth noting that multicodonic Leu, Ser, Pro, Ala, and Gly residues are the most common ones in high-frequency, low-complexity peptides whose function, in many cases, is the spacing of structural/functional domains [31]. Conversely, the mono/di-codonic amino acids Asn, Cys, Tyr, Met, Phe, and Trp are relatively rare in highly-frequent, low-complexity peptides and characterize functionally critical proteins such as proto-oncogenes [29]. In this case, specific usage of mono/di-codonic amino acids would allow the control of the proto-oncogene product at the transcriptional level. Moreover, during the last decade one of us proposed and demonstrated the association between rare pentapeptides and immunogenic potential [32-39]. Hence, understanding the mechanisms by which peptide platforms are used in the protein world not only is of biochemical interest but also proves of practical importance for biotechnology, e.g. vaccines, expression vectors and peptide therapy approaches [40] with the relevant advantage of effectiveness [41] without adverse side-effects [42-45].

## Authors' contributions

GC and CF performed the physico-(bio)chemical analyses; GN performed the standard nucleotide-nucleotide BLAST searches as reported in Table 1 and Additional file 1, Table S1; BT and MB performed the mathematical analyses and the statistical treatment of the data. AK performed the pentapeptide decomposition of the universal proteomes and determined the occurrence counts of the pentapeptides. DK proposed the original idea, interpreted the data, developed the research project, and wrote the manuscript. All authors read and approved the final manuscript.

## Supplementary Material

Additional file 1 **Table S1. The oligodeoxynucleotide sequences corresponding to never-expressed peptide motifs are mainly located in the non-coding strand**. The additional Table shows that the pentadecameric oligodeoxynucleotide sequences coding for the never-expressed pentapeptides correspond to untranslatable, frameshifted or mistranslated oligodeoxynucleotide sequences. Click here for file
