# Supplementary material for: The oligodeoxynucleotide sequences corresponding to never-expressed peptide motifs are mainly located in the non-coding strand
Source: BMC Bioinformatics. 2010 Jul 20;11:383. doi: 10.1186/1471-2105-11-383 (PMC2919516; doi:10.1186/1471-2105-11-383)
Supplement: Additional file 1 — Table S1. The oligodeoxynucleotide sequences corresponding to never-expressed peptide motifs are mainly located in the non-coding strand. The additional Table shows that the pentadecameric oligodeoxynucleotide sequences coding for the never-expressed pentapeptides correspond to untranslatable, frameshifted or mistranslated oligodeoxynucleotide sequences. [file 1471-2105-11-383-S1.DOC]

**Additional file 1**

**Table S1: The oligodeoxynucleotide sequences corresponding to never-expressed peptide motifs are mainly located in the non-coding strand**

| **AA sequence** | **Nucleotide sequences** | **Organisms** | **Total Occurrences** | **Location of the coding oligodeoxynucleotide:** |  |
| --- | --- | --- | --- | --- | --- |
|  |  |  |  | DNA minus strand | Other DNA Constraints |
| CDCMW | tgcgattgcatgtgg | 11 | 12 | 6 | 5 |
|  | tgtgattgcatgtgg | 8 | 9 | 3 | 6 |
|  | tgcgattgtatgtgg | 7 | 8 | 4 | 5 |
|  | tgtgattgtatgtgg | 12 | 13 | 8 | 5 |
|  | tgcgactgcatgtgg | 7 | 7 | 2 | 5 |
|  | tgtgactgcatgtgg | 7 | 9 | 4 | 5 |
|  | tgcgactgtatgtgg | 7 | 7 | 2 | 5 |
|  | tgtgactgtatgtgg | 8 | 9 | 5 | 4 |
| CECWY | tgcgaatgctggtat | 15 | 21 | 6 | 15 |
|  | tgtgaatgctggtat | 15 | 16 | 6 | 10 |
|  | tgcgagtgctggtat | 12 | 12 | 5 | 7 |
|  | tgcgaatgttggtat | 8 | 8 | 5 | 3 |
|  | tgcgaatgctggtac | 10 | 10 | 5 | 5 |
|  | tgtgagtgctggtat | 15 | 17 | 8 | 9 |
|  | tgcgagtgttggtat | 7 | 7 | 4 | 3 |
|  | tgcgaatgttggtac | 7 | 7 | 4 | 3 |
|  | tgtgaatgttggtat | 13 | 13 | 8 | 5 |
|  | tgcgagtgctggtac | 4 | 4 | 2 | 2 |
|  | tgtgagtgttggtat | 10 | 10 | 7 | 3 |
|  | tgcgagtgttggtac | 5 | 8 | 3 | 5 |
|  | tgtgaatgttggtac | 8 | 9 | 3 | 6 |
|  | tgtgaatgctggtac | 9 | 9 | 3 | 6 |
|  | tgtgagtgttggtac | 9 | 10 | 4 | 6 |
|  | tgtgagtgctggtac | 2 | 2 | 0 | 2 |
| CGMWW | tgcggcatgtggtgg | 24 | 26 | 17 | 9 |
|  | tgtggcatgtggtgg | 21 | 26 | 13 | 13 |
|  | tgcggaatgtggtgg | 14 | 14 | 7 | 7 |
|  | tgcgggatgtggtgg | 33 | 46 | 29 | 17 |
|  | tgtggaatgtggtgg | 22 | 25 | 9 | 16 |
|  | tgtgggatgtggtgg | 33 | 36 | 13 | 23 |
|  | tgcggtatgtggtgg | 15 | 15 | 10 | 5 |
|  | tgtggtatgtggtgg | 26 | 28 | 18 | 10 |
| CHWWC | tgccattggtggtgc | 52 | 69 | 37 | 32 |
|  | tgtcattggtggtgc | 49 | 54 | 29 | 25 |
|  | tgccactggtggtgc | 54 | 61 | 23 | 38 |
|  | tgccattggtggtgt | 39 | 47 | 27 | 20 |
|  | tgtcactggtggtgc | 44 | 50 | 21 | 29 |
|  | tgccactggtggtgt | 60 | 62 | 20 | 42 |
|  | tgtcattggtggtgt | 44 | 55 | 18 | 37 |
|  | tgtcactggtggtgt | 38 | 45 | 20 | 25 |
| CHWWM | tgccattggtggatg | 30 | 34 | 21 | 13 |
|  | tgtcattggtggatg | 20 | 21 | 7 | 14 |
|  | tgccactggtggatg | 37 | 44 | 25 | 19 |
|  | tgtcactggtggatg | 30 | 39 | 18 | 21 |
| CIWHW | tgcatttggcattgg | 33 | 46 | 26 | 20 |
|  | tgtatttggcattgg | 31 | 36 | 24 | 12 |
|  | tgcatatggcattgg | 17 | 20 | 9 | 11 |
|  | tgcatttggcactgg | 25 | 27 | 11 | 16 |
|  | tgtatatggcattgg | 20 | 27 | 14 | 13 |
|  | tgcatatggcactgg | 10 | 17 | 8 | 9 |
|  | tgtatttggcactgg | 17 | 19 | 7 | 12 |
|  | tgtatatggcactgg | 10 | 10 | 6 | 4 |
|  | tgtatctggcactgg | 19 | 20 | 10 | 10 |
|  | tgtatctggcattgg | 34 | 40 | 17 | 23 |
|  | tgcatctggcattgg | 24 | 29 | 15 | 14 |
|  | tgcatctggcactgg | 29 | 35 | 17 | 18 |
| CIWMW | tgcatttggatgtgg | 25 | 45 | 28 | 17 |
|  | tgtatttggatgtgg | 24 | 31 | 14 | 17 |
|  | tgcatctggatgtgg | 35 | 43 | 16 | 27 |
|  | tgcatatggatgtgg | 27 | 61 | 41 | 20 |
|  | tgtatctggatgtgg | 22 | 36 | 14 | 22 |
|  | tgtatatggatgtgg | 14 | 20 | 8 | 12 |
| CKCHW | tgcaaatgccattgg | 36 | 47 | 30 | 17 |
|  | tgcaaatgtcactgg | 16 | 23 | 9 | 14 |
|  | tgtaaatgtcattgg | 26 | 27 | 11 | 16 |
|  | tgcaagtgccactgg | 14 | 16 | 5 | 11 |
|  | tgtaaatgccactgg | 17 | 20 | 10 | 10 |
|  | tgcaagtgtcattgg | 15 | 17 | 7 | 10 |
|  | tgtaagtgtcactgg | 6 | 7 | 5 | 2 |
| CMCWM | tgcatgtgctggatg | 17 | 17 | 9 | 8 |
|  | tgtatgtgctggatg | 53 | 68 | 45 | 23 |
|  | tgcatgtgttggatg | 23 | 33 | 17 | 16 |
|  | tgtatgtgttggatg | 23 | 30 | 18 | 12 |
| CMHMW | tgcatgcatatgtgg | 25 | 39 | 21 | 18 |
|  | tgtatgcatatgtgg | 18 | 35 | 9 | 26 |
|  | tgcatgcacatgtgg | 10 | 47 | 16 | 21 |
|  | tgtatgcacatgtgg | 15 | 26 | 13 | 13 |
| CMMWQ | tgcatgatgtggcag | 24 | 28 | 17 | 11 |
|  | tgtatgatgtggcag | 21 | 23 | 13 | 10 |
|  | tgcatgatgtggcaa | 9 | 11 | 8 | 3 |
|  | tgtatgatgtggcaa | 19 | 20 | 12 | 8 |
| CMWAH | tgcatgtgggcgcat | 5 | 5 | 4 | 1 |
|  | tgtatgtgggcgcat | 6 | 8 | 3 | 5 |
|  | tgcatgtgggctcat | 6 | 8 | 6 | 2 |
|  | tgcatgtgggcgcac | 4 | 4 | 2 | 2 |
|  | tgcatgtgggcccat | 2 | 3 | 1 | 2 |
|  | tgcatgtgggcacat | 8 | 12 | 6 | 6 |
|  | tgcatgtgggctcac | 5 | 8 | 6 | 2 |
|  | tgtatgtgggcgcac | 6 | 6 | 3 | 3 |
|  | tgcatgtgggcccac | 8 | 12 | 9 | 3 |
|  | tgcatgtgggcacac | 11 | 13 | 9 | 4 |
|  | tgtatgtgggctcat | 6 | 6 | 2 | 4 |
|  | tgtatgtgggcccat | 5 | 7 | 4 | 3 |
|  | tgtatgtgggcacat | 13 | 15 | 9 | 6 |
|  | tgtatgtgggctcac | 6 | 6 | 4 | 2 |
|  | tgtatgtgggcccac | 2 | 2 | 1 | 1 |
|  | tgtatgtgggcacac | 10 | 10 | 6 | 4 |
| CQWWH | tgccagtggtggcat | 19 | 21 | 13 | 8 |
|  | tgtcagtggtggcat | 16 | 19 | 12 | 7 |
|  | tgccaatggtggcat | 38 | 45 | 24 | 21 |
|  | tgccagtggtggcac | 23 | 27 | 13 | 14 |
|  | tgtcaatggtggcat | 29 | 33 | 17 | 16 |
|  | tgtcagtggtggcac | 19 | 31 | 8 | 23 |
|  | tgccaatggtggcac | 35 | 49 | 27 | 22 |
|  | tgtcaatggtggcac | 19 | 25 | 15 | 10 |
| CWCMY | tgctggtgcatgtat | 12 | 19 | 10 | 9 |
|  | tgttggtgcatgtat | 18 | 33 | 16 | 17 |
|  | tgctggtgtatgtat | 17 | 27 | 19 | 8 |
|  | tgctggtgcatgtac | 24 | 40 | 30 | 10 |
|  | tgttggtgtatgtat | 27 | 48 | 31 | 17 |
|  | tgttggtgcatgtac | 13 | 24 | 15 | 9 |
|  | tgctggtgtatgtac | 15 | 17 | 9 | 8 |
|  | tgttggtgtatgtac | 15 | 20 | 11 | 9 |
| CWCWH | tgctggtgctggcat | 43 | 44 | 24 | 20 |
|  | tgttggtgctggcat | 28 | 56 | 40 | 16 |
|  | tgctggtgttggcat | 40 | 53 | 25 | 28 |
|  | tgctggtgctggcac | 42 | 50 | 26 | 24 |
|  | tgttggtgttggcat | 41 | 74 | 46 | 28 |
|  | tgttggtgctggcac | 58 | 65 | 40 | 25 |
|  | tgctggtgttggcac | 47 | 58 | 36 | 22 |
|  | tgttggtgttggcac | 41 | 52 | 29 | 23 |
| CWMWN | tgctggatgtggaac | 26 | 27 | 18 | 9 |
|  | tgttggatgtggaac | 34 | 38 | 13 | 25 |
|  | tgctggatgtggaat | 20 | 25 | 19 | 6 |
|  | tgttggatgtggaat | 23 | 28 | 15 | 13 |
| CWMWW | tgctggatgtggtgg | 20 | 21 | 10 | 11 |
|  | tgttggatgtggtgg | 37 | 38 | 20 | 18 |
| CWWHM | tgctggtggcatatg | 9 | 9 | 7 | 2 |
|  | tgttggtggcatatg | 13 | 13 | 6 | 7 |
|  | tgctggtggcacatg | 24 | 29 | 25 | 4 |
|  | tgttggtggcacatg | 13 | 14 | 6 | 8 |
| CWWMF | tgctggtggatgttt | 25 | 25 | 14 | 11 |
|  | tgttggtggatgttt | 24 | 24 | 15 | 9 |
|  | tgctggtggatgttc | 30 | 33 | 20 | 13 |
|  | tgttggtggatgttc | 5 | 5 | 5 | 0 |
| CYWMW | tgctattggatgtgg | 9 | 9 | 7 | 2 |
|  | tgttattggatgtgg | 41 | 45 | 23 | 22 |
|  | tgctactggatgtgg | 31 | 34 | 20 | 14 |
|  | tgttactggatgtgg | 26 | 26 | 10 | 16 |
| EWCMC | gaatggtgcatgtgc | 9 | 10 | 7 | 3 |
|  | gagtggtgcatgtgc | 12 | 12 | 8 | 4 |
|  | gaatggtgtatgtgc | 24 | 28 | 13 | 15 |
|  | gaatggtgcatgtgt | 11 | 16 | 9 | 7 |
|  | gagtggtgtatgtgc | 13 | 11 | 7 | 6 |
|  | gagtggtgcatgtgt | 10 | 10 | 6 | 4 |
|  | gagtggtgtatgtgt | 18 | 18 | 12 | 6 |
|  | gaatggtgtatgtgt | 17 | 23 | 10 | 13 |
| EWNCW | gaatggaactgctgg | 24 | 38 | 21 | 17 |
|  | gagtggaactgctgg | 17 | 28 | 19 | 9 |
|  | gaatggaattgctgg | 33 | 48 | 27 | 21 |
|  | gaatggaactgttgg | 20 | 22 | 10 | 12 |
|  | gagtggaattgctgg | 23 | 6 | 6 | 17 |
|  | gagtggaactgttgg | 10 | 16 | 6 | 10 |
|  | gaatggaattgttgg | 25 | 43 | 18 | 25 |
|  | gagtggaattgttgg | 38 | 24 | 23 | 15 |
| FWMWH | ttttggatgtggcat | 24 | 33 | 20 | 13 |
|  | ttctggatgtggcat | 44 | 50 | 13 | 37 |
|  | ttttggatgtggcac | 20 | 21 | 11 | 10 |
|  | ttctggatgtggcac | 23 | 22 | 15 | 7 |
| HCMYW | cattgcatgtattgg | 8 | 8 | 6 | 2 |
|  | cactgcatgtattgg | 13 | 14 | 6 | 8 |
|  | cattgtatgtattgg | 14 | 16 | 7 | 9 |
|  | cattgcatgtactgg | 6 | 6 | 3 | 3 |
|  | cactgtatgtattgg | 9 | 10 | 6 | 4 |
|  | cactgcatgtactgg | 8 | 9 | 7 | 2 |
|  | cattgtatgtactgg | 6 | 9 | 7 | 2 |
|  | cactgtatgtactgg | 13 | 13 | 7 | 6 |
| HMCWI | catatgtgctggatt | 13 | 14 | 8 | 6 |
|  | cacatgtgctggatt | 12 | 13 | 8 | 5 |
|  | catatgtgttggatt | 8 | 13 | 6 | 7 |
|  | catatgtgctggatc | 5 | 5 | 2 | 3 |
|  | catatgtgctggata | 21 | 21 | 5 | 16 |
|  | cacatgtgttggatt | 13 | 14 | 8 | 6 |
|  | cacatgtgctggatc | 13 | 14 | 10 | 4 |
|  | cacatgtgctggata | 7 | 9 | 3 | 6 |
|  | catatgtgttggatc | 13 | 13 | 9 | 4 |
|  | catatgtgttggata | 23 | 24 | 14 | 10 |
|  | cacatgtgttggatc | 2 | 3 | 3 | 0 |
|  | cacatgtgttggata | 9 | 11 | 6 | 5 |
| HMCWM | catatgtgctggatg | 14 | 16 | 8 | 8 |
|  | cacatgtgctggatg | 16 | 19 | 10 | 9 |
|  | catatgtgttggatg | 24 | 28 | 11 | 17 |
|  | cacatgtgttggatg | 14 | 18 | 5 | 13 |
| HMCWW | catatgtgctggtgg | 16 | 17 | 8 | 9 |
|  | cacatgtgctggtgg | 17 | 17 | 7 | 10 |
|  | catatgtgttggtgg | 11 | 14 | 9 | 5 |
|  | cacatgtgttggtgg | 15 | 18 | 11 | 7 |
| HMWCH | catatgtggtgccat | 14 | 16 | 10 | 6 |
|  | cacatgtggtgccat | 15 | 16 | 12 | 4 |
|  | catatgtggtgtcat | 22 | 22 | 8 | 14 |
|  | catatgtggtgccac | 11 | 13 | 2 | 11 |
|  | cacatgtggtgtcat | 14 | 17 | 13 | 4 |
|  | cacatgtggtgccac | 16 | 17 | 8 | 9 |
|  | catatgtggtgtcac | 6 | 6 | 3 | 3 |
|  | cacatgtggtgtcac | 5 | 7 | 2 | 5 |
| HWCNW | cattggtgcaactgg | 20 | 21 | 11 | 10 |
|  | cactggtgcaactgg | 30 | 60 | 42 | 18 |
|  | cattggtgtaactgg | 8 | 12 | 4 | 8 |
|  | cattggtgcaattgg | 28 | 40 | 12 | 28 |
|  | cactggtgtaactgg | 10 | 24 | 17 | 7 |
|  | cactggtgcaattgg | 17 | 25 | 14 | 11 |
|  | cattggtgtaattgg | 21 | 35 | 21 | 14 |
|  | cactggtgtaattgg | 12 | 13 | 4 | 9 |
| HWMCW | cattggatgtgctgg | 15 | 16 | 6 | 10 |
|  | cactggatgtgctgg | 38 | 59 | 32 | 27 |
|  | cattggatgtgttgg | 33 | 44 | 28 | 16 |
|  | cactggatgtgttgg | 27 | 39 | 16 | 23 |
| HYWWY | cattattggtggtat | 37 | 38 | 20 | 18 |
|  | cactattggtggtat | 17 | 20 | 7 | 13 |
|  | cattactggtggtat | 20 | 20 | 19 | 11 |
|  | cattattggtggtac | 24 | 41 | 29 | 12 |
|  | cactactggtggtat | 12 | 14 | 7 | 7 |
|  | cactattggtggtac | 11 | 21 | 5 | 16 |
|  | cattactggtggtac | 13 | 16 | 9 | 7 |
|  | cactactggtggtac | 19 | 23 | 12 | 11 |
| KWCWT | aaatggtgctggacc | 24 | 28 | 16 | 12 |
|  | aagtggtgctggacc | 29 | 36 | 11 | 25 |
|  | aaatggtgttggacc | 11 | 15 | 7 | 8 |
|  | aaatggtgctggact | 12 | 20 | 10 | 10 |
|  | aaatggtgctggaca | 41 | 57 | 32 | 25 |
|  | aaatggtgctggacg | 16 | 18 | 6 | 12 |
|  | aagtggtgctggact | 20 | 32 | 24 | 8 |
|  | aagtggtgctggaca | 33 | 50 | 22 | 28 |
|  | aagtggtgctggacg | 37 | 71 | 45 | 26 |
|  | aagtggtgttggacc | 13 | 16 | 8 | 8 |
|  | aaatggtgttggact | 21 | 29 | 11 | 18 |
|  | aaatggtgttggaca | 30 | 45 | 24 | 21 |
|  | aaatggtgttggacg | 8 | 13 | 2 | 11 |
|  | aagtggtgttggact | 12 | 17 | 11 | 6 |
|  | aagtggtgttggaca | 28 | 36 | 18 | 18 |
|  | aagtggtgttggacg | 15 | 16 | 8 | 8 |
| MCHWY | atgtgccattggtat | 16 | 21 | 15 | 6 |
|  | atgtgtcattggtat | 19 | 22 | 11 | 11 |
|  | atgtgccactggtat | 10 | 11 | 6 | 5 |
|  | atgtgccattggtac | 12 | 12 | 6 | 6 |
|  | atgtgtcactggtat | 8 | 8 | 4 | 4 |
|  | atgtgtcattggtac | 22 | 26 | 21 | 5 |
|  | atgtgccactggtac | 13 | 14 | 11 | 3 |
|  | atgtgtcactggtac | 23 | 23 | 4 | 19 |
| MCMWP | atgtgcatgtggccg | 23 | 28 | 21 | 7 |
|  | atgtgtatgtggccg | 11 | 14 | 6 | 8 |
|  | atgtgcatgtggcct | 8 | 8 | 3 | 5 |
|  | atgtgcatgtggccc | 22 | 24 | 13 | 11 |
|  | atgtgcatgtggcca | 24 | 27 | 12 | 15 |
|  | atgtgtatgtggcct | 9 | 13 | 6 | 7 |
|  | atgtgtatgtggccc | 13 | 15 | 3 | 12 |
|  | atgtgtatgtggcca | 22 | 23 | 12 | 11 |
| MCWWH | atgtgctggtggcat | 24 | 42 | 27 | 15 |
|  | atgtgttggtggcat | 27 | 29 | 11 | 18 |
|  | atgtgctggtggcac | 31 | 32 | 18 | 14 |
|  | atgtgttggtggcac | 10 | 10 | 5 | 5 |
| MCWWY | atgtgctggtggtat | 23 | 39 | 17 | 22 |
|  | atgtgttggtggtat | 21 | 21 | 5 | 16 |
|  | atgtgctggtggtac | 24 | 24 | 16 | 8 |
|  | atgtgttggtggtac | 11 | 12 | 5 | 7 |
| MHCWF | atgcattgctggttt | 18 | 31 | 11 | 20 |
|  | atgcactgctggttt | 20 | 21 | 14 | 7 |
|  | atgcattgttggttt | 30 | 34 | 14 | 20 |
|  | atgcattgctggttc | 14 | 14 | 8 | 6 |
|  | atgcactgttggttt | 13 | 15 | 6 | 9 |
|  | atgcactgctggttc | 16 | 17 | 7 | 10 |
|  | atgcattgttggttc | 10 | 13 | 4 | 9 |
|  | atgcactgttggttc | 7 | 7 | 1 | 6 |
| MMCNW | atgatgtgcaactgg | 27 | 35 | 24 | 11 |
|  | atgatgtgtaactgg | 12 | 13 | 9 | 4 |
|  | atgatgtgcaattgg | 19 | 20 | 8 | 12 |
|  | atgatgtgtaattgg | 10 | 12 | 5 | 7 |
| MMWYC | atgatgtggtattgc | 17 | 23 | 13 | 10 |
|  | atgatgtggtactgc | 18 | 31 | 22 | 9 |
|  | atgatgtggtattgt | 16 | 23 | 14 | 9 |
|  | atgatgtggtactgt | 7 | 8 | 5 | 3 |
|  | atgtggtgtatgtgt | 22 | 32 | 14 | 18 |
| MWCTM | atgtggtgcaccatg | 24 | 36 | 18 | 18 |
|  | atgtggtgtaccatg | 17 | 18 | 10 | 8 |
|  | atgtggtgcactatg | 18 | 21 | 12 | 9 |
|  | atgtggtgcacaatg | 26 | 38 | 22 | 16 |
|  | atgtggtgcacgatg | 23 | 25 | 13 | 12 |
|  | atgtggtgtactatg | 11 | 15 | 7 | 8 |
|  | atgtggtgtacaatg | 25 | 30 | 9 | 11 |
|  | atgtggtgtacgatg | 17 | 20 | 9 | 11 |
| MWHCW | atgtggcattgctgg | 29 | 45 | 22 | 23 |
|  | atgtggcactgctgg | 31 | 44 | 27 | 17 |
|  | atgtggcattgttgg | 19 | 24 | 14 | 10 |
|  | atgtggcactgttgg | 18 | 27 | 16 | 11 |
| MWHMC | atgtggcatatgtgc | 21 | 29 | 22 | 7 |
|  | atgtggcacatgtgc | 7 | 11 | 8 | 3 |
|  | atgtggcatatgtgt | 12 | 13 | 7 | 6 |
|  | atgtggcacatgtgt | 10 | 11 | 8 | 3 |
| MWHWW | atgtggcattggtgg | 32 | 55 | 34 | 21 |
|  | atgtggcactggtgg | 37 | 39 | 11 | 28 |
| MWWCW | atgtggtggtgctgg | 74 | 79 | 45 | 34 |
|  | atgtggtggtgttgg | 34 | 41 | 24 | 17 |
|  | atgtggtggatgcac | 39 | 32 | 13 | 19 |
| MWWNM | atgtggtggaacatg | 15 | 19 | 9 | 10 |
|  | atgtggtggaatatg | 44 | 47 | 25 | 22 |
| NMWMC | aacatgtggatgtgc | 16 | 18 | 7 | 11 |
|  | aatatgtggatgtgc | 22 | 26 | 11 | 15 |
|  | aacatgtggatgtgt | 22 | 27 | 11 | 16 |
|  | aatatgtggatgtgt | 57 | 54 | 48 | 6 |
| NWMWC | aactggatgtggtgc | 27 | 27 | 11 | 16 |
|  | aattggatgtggtgc | 16 | 19 | 7 | 12 |
|  | aactggatgtggtgt | 12 | 12 | 6 | 6 |
|  | aattggatgtggtgt | 19 | 19 | 11 | 9 |
| PCWWM | ccgtgctggtggatg | 67 | 70 | 38 | 32 |
|  | ccatgctggtggatg | 58 | 60 | 25 | 35 |
|  | ccctgctggtggatg | 55 | 60 | 28 | 32 |
|  | ccttgctggtggatg | 51 | 56 | 34 | 22 |
|  | ccatgttggtggatg | 28 | 31 | 14 | 17 |
|  | ccctgttggtggatg | 42 | 42 | 6 | 36 |
|  | ccttgttggtggatg | 36 | 39 | 16 | 23 |
|  | ccgtgttggtggatg | 21 | 23 | 10 | 13 |
| PWWCH | ccgtggtggtgccat | 33 | 34 | 25 | 9 |
|  | ccttggtggtgccat | 43 | 49 | 24 | 25 |
|  | ccatggtggtgccat | 35 | 42 | 20 | 22 |
|  | ccctggtggtgccat | 26 | 34 | 17 | 17 |
|  | ccgtggtggtgtcat | 26 | 36 | 23 | 13 |
|  | ccgtggtggtgccac | 36 | 51 | 32 | 19 |
|  | ccttggtggtgtcat | 26 | 36 | 20 | 16 |
|  | ccatggtggtgtcat | 29 | 35 | 22 | 13 |
|  | ccctggtggtgtcat | 19 | 23 | 11 | 12 |
|  | ccttggtggtgccac | 34 | 48 | 29 | 19 |
|  | ccatggtggtgccac | 26 | 38 | 15 | 23 |
|  | ccctggtggtgccac | 22 | 30 | 14 | 16 |
|  | ccgtggtggtgtcac | 37 | 43 | 21 | 22 |
|  | ccttggtggtgtcac | 30 | 43 | 28 | 15 |
|  | ccatggtggtgtcac | 18 | 19 | 7 | 12 |
|  | ccctggtggtgtcac | 18 | 21 | 11 | 10 |
| QWCCM | cagtggtgctgcatg | 15 | 18 | 12 | 6 |
|  | caatggtgctgcatg | 30 | 33 | 15 | 18 |
|  | cagtggtgttgcatg | 21 | 24 | 19 | 5 |
|  | cagtggtgctgtatg | 13 | 19 | 2 | 17 |
|  | caatggtgttgcatg | 15 | 17 | 9 | 8 |
|  | caatggtgctgtatg | 11 | 11 | 5 | 6 |
|  | cagtggtgttgtatg | 18 | 21 | 12 | 9 |
|  | caatggtgttgtatg | 20 | 22 | 12 | 10 |
| QWCWM | cagtggtgctggatg | 29 | 30 | 14 | 16 |
|  | caatggtgctggatg | 32 | 33 | 14 | 19 |
|  | cagtggtgttggatg | 19 | 20 | 7 | 13 |
|  | caatggtgttggatg | 19 | 21 | 14 | 7 |
| QWMWW | cagtggatgtggtgg | 30 | 37 | 18 | 19 |
|  | caatggatgtggtgg | 29 | 36 | 18 | 18 |
| RCWMM | cgctgctggatgatg | 59 | 62 | 40 | 22 |
|  | agctgctggatgatg | 31 | 33 | 15 | 18 |
|  | cgttgctggatgatg | 78 | 83 | 32 | 51 |
|  | cgatgctggatgatg | 30 | 33 | 13 | 20 |
|  | cggtgctggatgatg | 56 | 57 | 20 | 37 |
|  | cgctgttggatgatg | 19 | 23 | 9 | 14 |
|  | agttgctggatgatg | 69 | 76 | 44 | 32 |
|  | agatgctggatgatg | 56 | 62 | 19 | 43 |
|  | aggtgctggatgatg | 50 | 62 | 31 | 31 |
|  | agctgttggatgatg | 33 | 36 | 13 | 25 |
|  | cgttgttggatgatg | 26 | 34 | 20 | 14 |
|  | cgatgttggatgatg | 21 | 33 | 5 | 28 |
|  | cggtgttggatgatg | 24 | 31 | 12 | 19 |
|  | agttgttggatgatg | 35 | 42 | 16 | 27 |
|  | agatgttggatgatg | 32 | 37 | 13 | 24 |
|  | aggtgttggatgatg | 40 | 56 | 35 | 21 |
| TMWMW | accatgtggatgtgg | 37 | 39 | 24 | 15 |
|  | actatgtggatgtgg | 34 | 41 | 13 | 28 |
|  | acaatgtggatgtgg | 30 | 37 | 13 | 24 |
|  | acgatgtggatgtgg | 42 | 53 | 20 | 33 |
| VMCWH | gtgatgtgctggcat | 13 | 15 | 10 | 5 |
|  | gtaatgtgctggcat | 7 | 8 | 7 | 1 |
|  | gtcatgtgctggcat | 5 | 5 | 3 | 2 |
|  | gttatgtgctggcat | 6 | 6 | 1 | 5 |
|  | gtgatgtgttggcat | 24 | 25 | 12 | 13 |
|  | gtgatgtgctggcac | 25 | 27 | 19 | 8 |
|  | gtaatgtgttggcat | 11 | 13 | 6 | 7 |
|  | gtcatgtgttggcat | 10 | 11 | 4 | 7 |
|  | gttatgtgttggcat | 11 | 8 | 5 | 6 |
|  | gtaatgtgctggcac | 8 | 10 | 4 | 6 |
|  | gtcatgtgctggcac | 8 | 9 | 3 | 6 |
|  | gttatgtgctggcac | 9 | 11 | 3 | 8 |
|  | gtgatgtgttggcac | 7 | 7 | 3 | 4 |
|  | gtaatgtgttggcac | 9 | 14 | 11 | 3 |
|  | gtcatgtgttggcac | 13 | 15 | 7 | 8 |
|  | gttatgtgttggcac | 9 | 9 | 3 | 6 |
| WCMMW | tggtgcatgatgtgg | 25 | 33 | 25 | 8 |
|  | tggtgtatgatgtgg | 36 | 33 | 8 | 25 |
| WCQHM | tggtgccagcatatg | 14 | 23 | 14 | 9 |
|  | tggtgtcagcatatg | 6 | 6 | 5 | 1 |
|  | tggtgccaacatatg | 13 | 14 | 10 | 4 |
|  | tggtgccagcacatg | 11 | 12 | 1 | 11 |
|  | tggtgtcaacatatg | 7 | 8 | 4 | 4 |
|  | tggtgtcagcacatg | 12 | 12 | 5 | 7 |
|  | tggtgccaacacatg | 15 | 15 | 8 | 7 |
|  | tggtgtcaacacatg | 14 | 15 | 6 | 9 |
| WCWMY | tggtgctggatgtat | 31 | 45 | 28 | 17 |
|  | tggtgttggatgtat | 18 | 18 | 11 | 7 |
|  | tggtgctggatgtac | 38 | 38 | 15 | 23 |
|  | tggtgttggatgtac | 9 | 9 | 4 | 5 |
| WCYHM | tggtgctatcatatg | 15 | 22 | 11 | 11 |
|  | tggtgttatcatatg | 9 | 11 | 4 | 7 |
|  | tggtgctaccatatg | 10 | 11 | 7 | 4 |
|  | tggtgctatcacatg | 10 | 13 | 8 | 5 |
|  | tggtgttaccatatg | 6 | 7 | 1 | 6 |
|  | tggtgttatcacatg | 7 | 7 | 2 | 5 |
|  | tggtgctaccacatg | 12 | 13 | 4 | 9 |
|  | tggtgttaccacatg | 9 | 10 | 6 | 4 |
| WFQCM | tggtttcagtgcatg | 19 | 34 | 27 | 7 |
|  | tggttccagtgcatg | 13 | 14 | 8 | 6 |
|  | tggtttcaatgcatg | 19 | 23 | 16 | 7 |
|  | tggtttcagtgtatg | 22 | 26 | 9 | 17 |
|  | tggttccaatgcatg | 9 | 9 | 5 | 4 |
|  | tggttccagtgtatg | 9 | 11 | 7 | 4 |
|  | tggttccaatgtatg | 17 | 17 | 17 | 0 |
|  | tggtttcaatgtatg | 18 | 23 | 11 | 12 |
| WHCCM | tggcattgctgcatg | 27 | 35 | 25 | 10 |
|  | tggcactgctgcatg | 18 | 19 | 9 | 10 |
|  | tggcattgttgcatg | 25 | 29 | 18 | 11 |
|  | tggcattgctgtatg | 19 | 22 | 16 | 6 |
|  | tggcactgttgcatg | 24 | 26 | 12 | 14 |
|  | tggcactgctgtatg | 15 | 19 | 9 | 10 |
|  | tggcattgttgtatg | 30 | 31 | 17 | 14 |
|  | tggcactgttgtatg | 7 | 8 | 2 | 6 |
| WHIMW | tggcatattatgtgg | 12 | 18 | 10 | 8 |
|  | tggcacattatgtgg | 14 | 21 | 14 | 7 |
|  | tggcatatcatgtgg | 17 | 17 | 11 | 6 |
|  | tggcatataatgtgg | 10 | 11 | 6 | 5 |
|  | tggcacatcatgtgg | 11 | 13 | 7 | 6 |
|  | tggcacataatgtgg | 9 | 10 | 6 | 4 |
| WMCHM | tggatgtgccatatg | 11 | 14 | 10 | 4 |
|  | tggatgtgtcatatg | 18 | 21 | 5 | 16 |
|  | tggatgtgccacatg | 21 | 23 | 12 | 11 |
|  | tggatgtgtcacatg | 14 | 20 | 10 | 10 |
| WMCHW | tggatgtgccattgg | 43 | 47 | 30 | 17 |
|  | tggatgtgtcattgg | 21 | 30 | 17 | 13 |
|  | tggatgtgccactgg | 34 | 40 | 24 | 16 |
|  | tggatgtgtcactgg | 27 | 30 | 16 | 14 |
| WMCNW | tggatgtgcaactgg | 29 | 29 | 16 | 13 |
|  | tggatgtgtaactgg | 10 | 13 | 7 | 6 |
|  | tggatgtgcaattgg | 59 | 68 | 34 | 34 |
|  | tggatgtgtaattgg | 21 | 25 | 16 | 9 |
| WMCWF | tggatgtgctggttt | 26 | 48 | 28 | 20 |
|  | tggatgcgctggttt | 28 | 31 | 12 | 19 |
|  | tggatgtgctggttc | 29 | 37 | 23 | 14 |
|  | tggatgcgctggttc | 49 | 50 | 14 | 36 |
| WMQCW | tggatgcagtgctgg | 40 | 47 | 26 | 21 |
|  | tggatgcaatgctgg | 60 | 78 | 34 | 44 |
|  | tggatgcagtgttgg | 32 | 46 | 34 | 12 |
|  | tggatgcaatgttgg | 26 | 52 | 39 | 13 |
| WWHCH | tggtggcattgccat | 42 | 58 | 43 | 15 |
|  | tggtggcactgccat | 40 | 43 | 24 | 19 |
|  | tggtggcattgtcat | 49 | 57 | 31 | 26 |
|  | tggtggcattgccac | 34 | 37 | 14 | 23 |
|  | tggtggcactgtcat | 37 | 41 | 20 | 21 |
|  | tggtggcactgccac | 41 | 43 | 18 | 25 |
|  | tggtggcattgtcac | 40 | 48 | 27 | 21 |
|  | tggtggcactgtcac | 36 | 38 | 21 | 17 |
| WWHCW | tggtggcattgctgg | 19 | 24 | 13 | 11 |
|  | tggtggcactgctgg | 67 | 68 | 31 | 37 |
|  | tggtggcattgttgg | 50 | 58 | 29 | 29 |
|  | tggtggcactgttgg | 43 | 46 | 23 | 23 |
| WWHHC | tggtggcatcattgc | 44 | 57 | 30 | 27 |
|  | tggtggcaccattgc | 33 | 42 | 24 | 18 |
|  | tggtggcatcactgc | 30 | 36 | 15 | 21 |
|  | tggtggcatcattgt | 39 | 44 | 27 | 17 |
|  | tggtggcaccactgc | 23 | 37 | 14 | 23 |
|  | tggtggcaccattgt | 22 | 29 | 20 | 9 |
|  | tggtggcatcactgt | 31 | 33 | 17 | 16 |
|  | tggtggcaccactgt | 24 | 32 | 22 | 10 |
| WWHMC | tggtggcatatgtgc | 13 | 19 | 14 | 5 |
|  | tggtggcacatgtgc | 15 | 16 | 9 | 7 |
|  | tggtggcatatgtgt | 12 | 12 | 5 | 7 |
|  | tggtggcacatgtgt | 24 | 34 | 8 | 26 |
| WWMHM | tggtggatgcatatg | 12 | 16 | 8 | 8 |
|  | tggtggatgcacatg | 19 | 21 | 10 | 11 |
| WWYCW | tggtggtattgctgg | 60 | 66 | 30 | 36 |
|  | tggtggtactgctgg | 58 | 60 | 47 | 13 |
|  | tggtggtattgttgg | 57 | 66 | 26 | 40 |
|  | tggtggtactgttgg | 57 | 65 | 35 | 30 |
| WYPCW | tggtatccgtgctgg | 33 | 33 | 19 | 14 |
|  | tggtacccgtgctgg | 15 | 24 | 12 | 12 |
|  | tggtatccttgctgg | 39 | 52 | 16 | 36 |
|  | tggtatccatgctgg | 29 | 34 | 12 | 22 |
|  | tggtatccctgctgg | 26 | 45 | 30 | 15 |
|  | tggtatccgtgttgg | 23 | 26 | 12 | 14 |
|  | tggtacccttgctgg | 19 | 23 | 12 | 11 |
|  | tggtacccatgctgg | 17 | 21 | 10 | 11 |
|  | tggtacccctgctgg | 17 | 19 | 9 | 10 |
|  | tggtacccgtgttgg | 6 | 7 | 2 | 5 |
|  | tggtatccttgttgg | 25 | 27 | 15 | 12 |
|  | tggtatccatgttgg | 24 | 27 | 12 | 15 |
|  | tggtatccctgttgg | 27 | 33 | 4 | 29 |
|  | tggtacccttgttgg | 7 | 13 | 12 | 1 |
|  | tggtacccatgttgg | 15 | 19 | 10 | 9 |
|  | tggtacccctgttgg | 4 | 5 | 1 | 4 |
| YHWCM | tatcattggtgcatg | 10 | 12 | 6 | 6 |
|  | taccattggtgcatg | 9 | 13 | 8 | 5 |
|  | tatcactggtgcatg | 9 | 10 | 6 | 4 |
|  | tatcattggtgtatg | 10 | 17 | 12 | 5 |
|  | taccactggtgcatg | 12 | 16 | 6 | 10 |
|  | taccattggtgtatg | 9 | 14 | 7 | 7 |
|  | tatcactggtgtatg | 20 | 32 | 16 | 16 |
|  | taccactggtgtatg | 11 | 55 | 24 | 31 |
| YMCHW | tatatgtgccattgg | 22 | 25 | 16 | 9 |
|  | tacatgtgccattgg | 6 | 6 | 1 | 5 |
|  | tatatgtgtcattgg | 6 | 26 | 13 | 13 |
|  | tatatgtgccactgg | 6 | 9 | 6 | 3 |
|  | tacatgtgtcattgg | 6 | 11 | 8 | 3 |
|  | tacatgtgccactgg | 6 | 6 | 2 | 4 |
|  | tatatgtgtcactgg | 9 | 9 | 6 | 3 |
|  | tacatgtgtcactgg | 1 | 1 | 0 | 1 |
| YWWMC | tattggtggatgtgc | 40 | 43 | 13 | 30 |
|  | tactggtggatgtgc | 11 | 12 | 6 | 6 |
|  | tattggtggatgtgt | 4 | 52 | 32 | 20 |
|  | tactggtggatgtgt | 10 | 17 | 8 | 9 |

Pentapeptide sequences were retrotranslated into the corresponding coding oligodeoxynucleotide sequences. For each pentapeptide under analysis, the most likely, optimized oligodeoxynucleotide coding frame and the degenerate ones were considered. Then, each pentadecameric oligodeoxynucleotide sequence was used as a probe to scan the entire NCBI nucleotide collection for exact pentadecameric matches using the BLAST (blastn) program with no gaps allowed. Underlined sequences refer to the oligodeoxynucleotide sequences obtained using the Sequence Manipulation Suite Reverse Translate program (http://www.bioinformatics.org/sms2/) [14] that generates the most likely coding sequence. Additionally, Reverse Translate a Protein (http://www.vivo.colostate.edu/molkit/rtranslate/index.html), a program that uses the standard genetic code and does not consider differences in codon usage, was used in order to obtain all the possible oligodeoxynucleotide coding frames for each pentapeptide under analysis.

The columns in the Table refer, in the order, to: sequence of the never-expressed pentapeptides in one letter amino acid code; corresponding possible oligodeoxynucleotide coding frames with the most likely one underlined; number of organisms hosting oligodeoxynucleotide sequences; total number of oligodeoxynucleotide sequence occurrences; DNA location of the oligodeoxynucleotide sequences. Other DNA constraints refer to introns, pseudogenes, frameshifts, and untranslated regions (UTRs).
